# Mydriasis associated with ischemic cerebrovascular infarct affecting the ipsilateral cerebellar interposital nucleus in 2 dogs

**DOI:** 10.1111/jvim.17176

**Published:** 2024-08-28

**Authors:** Cecilia‐Gabriella Danciu, Joe Fenn, Elsa Beltran

**Affiliations:** ^1^ Department of Clinical Science and Services, Royal Veterinary College University of London Hatfield UK

**Keywords:** anisocoria, canine, cerebellar nuclei, ischemic stroke

## Abstract

A 10‐year‐old male neutered crossbreed dog and an 8‐year‐old female neutered greyhound presented after peracute onset of cerebellar dysfunction. The crossbreed dog had anisocoria with the left pupil being mydriatic, spontaneous conjugate rotatory nystagmus with fast phase to the left and delayed postural reactions on the left side. The greyhound had anisocoria with the left pupil being mydriatic, right positional ventral strabismus, absent menace response in the left eye and postural reaction deficits on the left side. For both dogs, the neuroanatomical localization was left cerebellum with paradoxical vestibular syndrome. Magnetic resonance imaging identified a left cerebellar ischemic territorial infarct of the rostral cerebellar artery, involving the region of the left interposital nucleus. Both dogs were given supportive care and at 2‐week follow‐up the anisocoria had resolved. Anisocoria with mydriasis can be a clinical sign in dogs with naturally‐occurring cerebellar ischemic infarcts in the region of the ipsilateral interposital nucleus.

**Abbreviations**
DWI: diffusion‐weighted imaging
FLAIR: fluid attenuated inversion recovery
MRI: magnetic resonance imaging
PLR: pupillary light reflex
RI: reference interval

## 1 INTRODUCTION

Anisocoria is the occurrence of unequal pupil size and can be physiological, pathological, or pharmacological in origin.^1^ Pathological anisocoria can be the result of a primary ophthalmologic disorder, such as corneal, iris, lens or retinal conditions, and increased in intraocular pressure.^1^ Neurological causes of anisocoria include lesions affecting the optic and oculomotor nerves, as well as the sympathetic innervation to the eyes.^1^, ^2^ Pharmacological anisocoria can be caused by plant alkaloid intoxication (*Datura stramonium*),^3^ topically applied parasympathomimetic eye drops (eg, pilocarpine) used to constrict the pupil,^1^, ^2^, ^4^ or topical sympathomimetic (eg, phenylephrine) eye drops used to dilate the pupil.^1^

Experimentally, anisocoria also has been reported after ablation of the fastigial and interposital nuclei of the cerebellum in cats.^5^ Ablation of the fastigial nucleus caused contralateral mydriasis with an incomplete pupillary light reflex (PLR) and ipsilateral third eyelid protrusion. Unilateral ablation of the interposital nucleus caused ipsilateral mydriasis, incomplete PLR, an ipsilateral widening of the palpebral fissure, and a contralateral elevation of the third eyelid.^5^ Clinically, neurological conditions causing anisocoria in dogs and cats include Horner syndrome,^6^ idiopathic oculomotor neuropathy,^7^ Pourfour du Petit syndrome,^8^ and asymmetrical midbrain lesions affecting the parasympathetic nucleus of the oculomotor nerve.^9^

Anisocoria has been reported sporadically in cases with cerebellar infarcts but has not previously been discussed thoroughly.^10^, ^11^ Here, we describe the clinical presentation, diagnostic findings and outcome of 2 dogs with ipsilateral mydriasis presumed to be associated with ischemic infarcts affecting the cerebellar interposital nucleus.

## 2 CASE DESCRIPTION

### 2.1 Case 1

**FIGURE 1:**
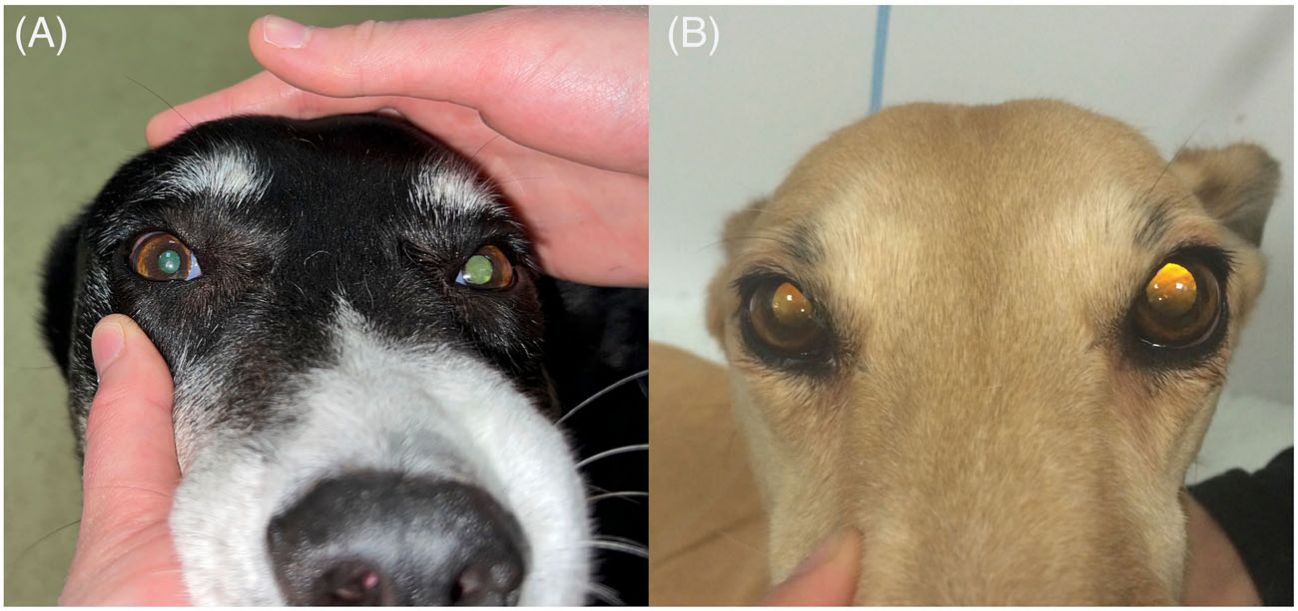
Images illustrating anisocoria with the left pupil being mydriatic in the crossbreed (A) and greyhound (B) during stimulation with a source of bright light.

A 10‐year‐10‐months old male neutered crossbreed dog was presented with peracute onset of ataxia, which was preceded by vomiting. Abnormalities were not detected on physical examination. Neurological examination indicated appropriate mentation, and the dog was nonambulatory because of severe vestibulocerebellar ataxia. Cranial nerve examination showed a right‐sided head tilt, anisocoria, with a mydriatic left pupil and incomplete PLR (Figure 1A). Spontaneous conjugate rotatory nystagmus with a fast phase to the left and positional ventral strabismus in the right eye were present. Postural reactions were intact in the right thoracic and pelvic limbs but delayed in the left thoracic and pelvic limbs. Spinal reflexes all were intact with increased tone in the left thoracic and pelvic limbs, and no pain was detected on spinal or cranial palpation. Neuroanatomic localization was to the left cerebellum with paradoxical vestibular syndrome.

**FIGURE 2:**
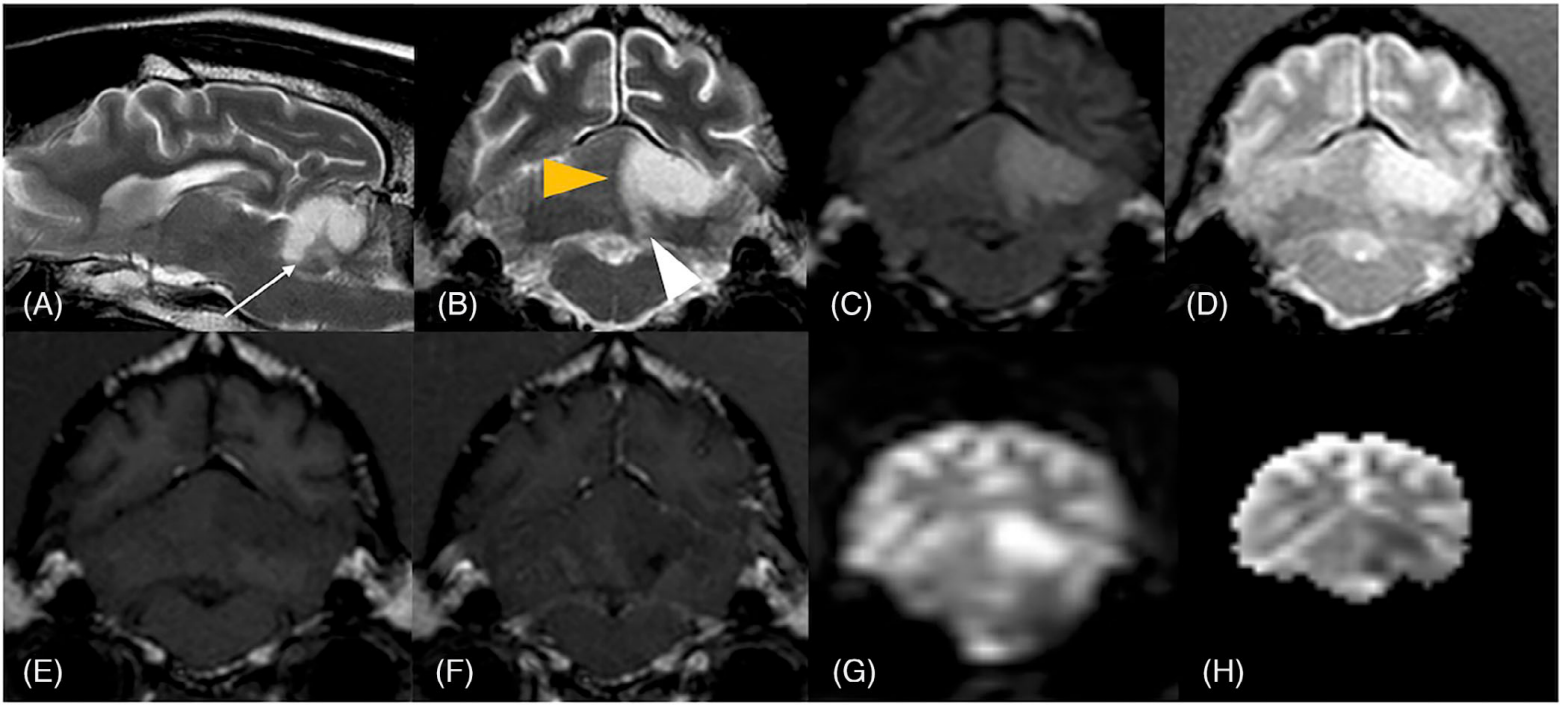
Magnetic resonance imaging of the brain of a 10‐year‐10‐months old male neutered crossbreed dog with peracute onset of left cerebellar dysfunction. On T2‐weighted (w) sagittal (A), T2‐w transverse (B) and fluid attenuated inversion recovery (C) imaging, the territory of the left rostral cerebellar artery (white arrows) including the region of the interposital nucleus (white arrowhead) is hyperintense and swollen causing mild midline shift to the right (yellow arrowhead). This region is hypointense in T1‐w transverse (E) image without contrast enhancement of the cerebellar cortex and meninges (F). The same lesion appears hyperintense on gradient echo sequence without evidence of signal void (D). On diffusion‐weighted images (G) there is hyperintense signal corresponding to the same area seen on T2‐w images and on the apparent diffusion coefficient map (H) there is a corresponding area of hypointensity indicating restricted diffusion.

Complete blood count was within normal limits. Serum biochemistry disclosed mild hypernatremia (155.2 mEq/L; reference interval [RI], 135‐155 mEq/L), moderately increased creatine kinase activity (9593 U/L; RI, 67‐446 U/L), and mildly increased C‐reactive protein concentration (29.3 mg/L; RI, <10 mg/L). Serum total thyroxine concentration was 0.61 μg/dL (RI, 1‐4.03 μg/dL) with normal thyroid‐stimulating hormone (0.10 ng/mL; RI, <0.41 ng/mL) consistent with euthyroid sick syndrome. Urinalysis results were normal, including urine protein: creatinine ratio. The dog was normotensive on noninvasive blood pressure measurements. Ophthalmic examination was normal, except for anisocoria, incomplete PLR, and rotatory nystagmus. Magnetic resonance imaging (MRI) of the head performed using a 1.5 T unit (Intera 1.5 T, Philips Healthcare, Eindhoven, the Netherlands) under general anesthesia identified a left‐sided, sharply demarcated, wedge‐shaped T2‐weighted and fluid attenuated inversion recovery (FLAIR) hyperintense and T1‐weighted hypointense lesion in the left cerebellar hemisphere, with minimal to no mass effect, no contrast enhancement and restricted diffusion on diffusion‐weighted imaging (DWI). The lesion included the region of the left interposital nucleus (Figure 2). Based on the clinical presentation and diagnostic findings, a presumptive diagnosis of left rostral cerebellar territorial ischemic infarct was made. Computed tomography scan (Aquilion ONE Genesis 320 slice, Canon Medical System, Otawara, Japan) of the thoracic and abdominal cavities identified no evidence of underlying causes.

The dog received supportive care, consisting of administration of maropitant (1 mg/kg IV q24h), assisted feeding, manual turning, and physical therapy. The dog was discharged after 4 days with mild vestibular ataxia, falling to the right side, right head tilt, and improved but incomplete PLR in the left eye. Reexamination 2 weeks after discharge showed marked improvement with only mild right head tilt, resolved anisocoria, and mild cerebellar ataxia with hypermetric gait in the left thoracic and pelvic limbs. On follow‐up telephone consultation 6 months after diagnosis, the dog was reportedly neurologically normal.

### 2.2 Case 2

An 8‐year‐old female spayed greyhound was presented with a history of peracute onset of inability to stand, with weakness on the left side. Abnormalities were not detected on physical examination. Neurological examination showed appropriate mentation, left lateralizing nonambulatory tetraparesis, and intermittent opisthotonus. Cranial nerve examination identified right‐sided head tilt, anisocoria with the left pupil being mydriatic, and normal PLR (Figure 1B). The menace response was absent in the left eye with normal vision, with intact menace response and vision in the right eye. Right positional ventral strabismus was observed with no evidence of spontaneous nystagmus. The left palpebral fissure was subjectively wider than the right. Postural reactions were delayed in the left thoracic and pelvic limbs. Spinal reflexes were normal, and no pain was elicited on spinal or cranial palpation. Neuroanatomical localization was to the left cerebellum, with paradoxical vestibular syndrome.

**FIGURE 3:**
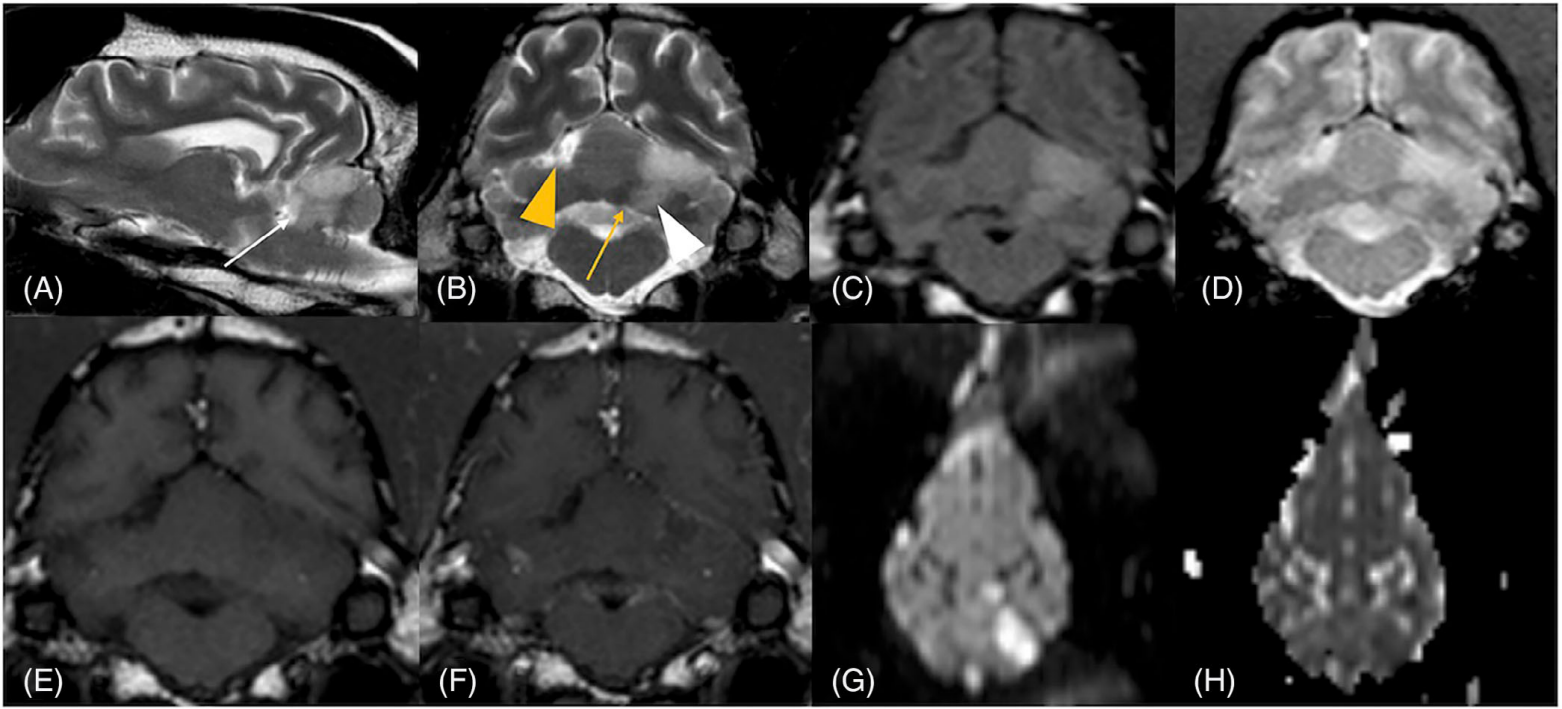
Magnetic resonance imaging of the brain of a 8‐year‐old female spayed greyhound with a peracute onset of left cerebellar dysfunction. On T2‐weighted (w) sagittal (A) and T2‐w transverse (B) and fluid attenuated inversion recovery transverse (C) images, the territory of the left rostral cerebellar artery (white arrow) including the region of the interposital nucleus (white arrowhead) is hyperintense compared to gray matter and there is mild swelling indicating mild midline shift (yellow arrow). The same region is hypointense in T1‐w pre‐ (E) and postcontrast transverse (F) images with no evidence of contrast enhancement of the cerebellar cortex and meninges (F). The same lesion appears hyperintense on gradient echo transverse sequence without evidence of signal void (D). On dorsal diffusion‐weighted images (G) there is hyperintense signal corresponding to the same area seen on T2‐w images, and on the dorsal apparent diffusion coefficient map (H) there is a corresponding area of hypointensity indicating restricted diffusion. Additionally, a T2‐w transverse (B) hyperintense, fluid attenuated inversion recovery (C) and T1‐w (E) hypointense lesion with no contrast enhancement (F) is present in the right rostral cerebellum (yellow arrowhead). This area does not have restricted diffusion (G and H).

**FIGURE 4:**
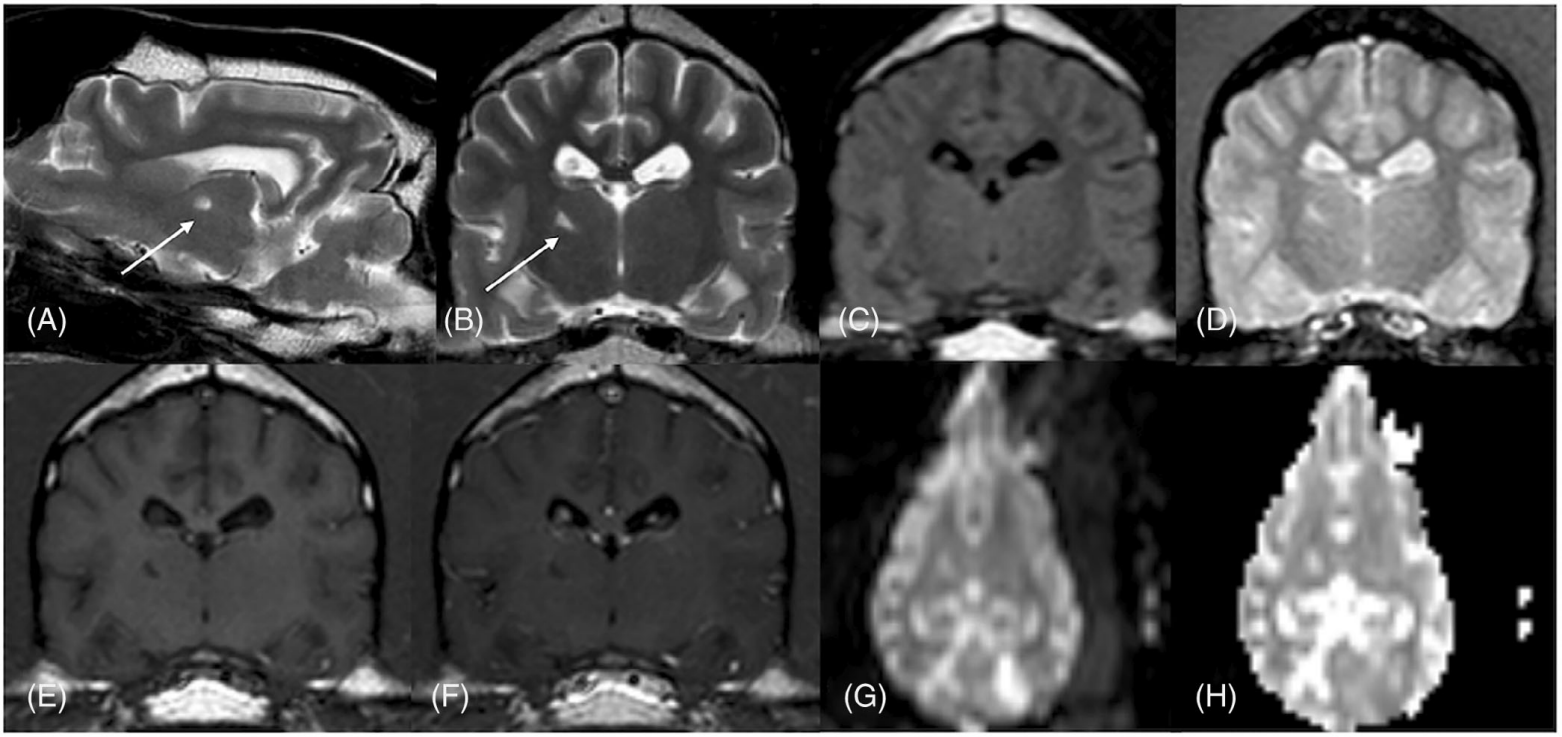
Magnetic resonance imaging of the brain of a 8‐year‐old female spayed greyhound with a peracute onset of left cerebellar dysfunction. On the T2‐weighted (w) sagittal (A) and T2‐w transverse (B) and fluid attenuated inversion recovery transverse (C) images, the territory of the distal perforating artery of the right thalamus (white arrow) is hyperintense compared to gray matter. The same region appears hyperintense on gradient echo transverse sequence without evidence of signal void (D), hypointense in T1w pre‐ (E) and postcontrast (F) transverse images with no contrast enhancement and no restricted diffusion in dorsal diffusion‐weighted images (G) and dorsal apparent diffusion coefficient map (H).

Abnormalities were not detected on CBC and noninvasive blood pressure measurements. Serum biochemistry identified mildly increased serum creatinine concentration of 1.93 mg/dL (RI, 0.66‐1.6 mg/dL) and creatine kinase activity of 516 U/L (RI, 61‐394 U/L). Urinalysis was normal, but the urine protein: creatinine ratio was increased at 1.96 (RI, 0‐0.5). Magnetic resonance imaging of the head was performed using a 1.5 T unit (Intera 1.5 T, Philips Healthcare, Eindhoven, the Netherlands) with the dog under general anesthesia. Magnetic resonance imaging identified a left‐sided, sharply demarcated, wedge‐shaped T2‐weighted and FLAIR hyperintense and T1‐weighted hypointense lesion in the left cerebellar hemisphere, with minimal contrast enhancement and minimal mass effect. The lesion showed restricted diffusion on DWI sequences (Figure 3). As in Case 1, the lesion included the region of the left interposital nucleus. A small, focal T2‐weighted hyper‐ and T1‐weighted hypointense lesion in the right thalamus also was identified with no contrast enhancement (Figure 4). Additionally, in the right rostral cerebellum, a triangular shaped T2‐weighted hyper‐, FLAIR hypo‐, and T1‐weighted hypointense lesion was identified with no restricted diffusion or contrast uptake. The latter lesion had no extension toward the deep cerebellar nuclei. At the level of the left caudal colliculus, a focal, ill‐defined T2‐weighted and FLAIR hyperintense lesion, T1‐weighted iso on pre‐ and postcontrast sequences with no mass effect or restricted diffusion was identified (Figure 3). Based on these findings, a presumed left‐sided rostral cerebellar territorial and lacunar right thalamic ischemic infarct was diagnosed, with an old right‐sided cerebellar territorial and left‐sided lacunar ischemic infarct at the level of the caudal colliculus. The chronic and acute ischemic infarcts may have been associated with protein‐losing nephropathy, based on the increased urine protein: creatinine ratio. Abnormalities were not detected on thoracic radiography and abdominal ultrasound examination.

The dog received supportive care, consisting of assisted feeding, manual turning, and physical rehabilitation. At discharge 7 days later the dog was ambulatory with improved cerebellovestibular ataxia, and mild right‐sided head tilt. The referring veterinarian continued management of the protein‐losing nephropathy.

At 2‐week reexamination after discharge, neurological signs were improving with mild right‐sided head tilt present and resolved anisocoria. On follow‐up telephone consultation 4 months after diagnosis, the dog was neurologically normal. Three years later the dog was euthanized because of suspected humeral osteosarcoma.

## 3 DISCUSSION

We describe the occurrence of anisocoria characterized by mydriasis associated with naturally‐occurring cerebellar ischemic infarcts in the region of the ipsilateral interposital nucleus in 2 dogs. Magnetic resonance imaging findings were consistent with an ischemic infarct in the territory of the left rostral cerebellar artery in both dogs, as well as ischemic infarction in the vascular territory of the distal perforating artery of the right thalamus in the greyhound. In both cases the anisocoria resolved over a period of 2 weeks, with complete resolution of cerebellovestibular signs within 4 to 6 months.

Pupil size is regulated by the sympathetic and parasympathetic nervous system in response to light in the environment and the emotional status of the patient.^1^, ^2^ Parasympathetic innervation of the eye regulates the response to environmental light with innervation originating from the parasympathetic nucleus of the oculomotor nerve in the midbrain.^1^, ^2^ The preganglionic parasympathetic fibers course rostrally through the orbital fissure into the ciliary ganglion, forming the short ciliary nerves and innervating the ciliary muscle and the sphincter of the pupil.^1^, ^12^ Any lesion along this pathway can result in parasympathetic dysfunction, clinically observed as mydriasis, and the patient will have a partial or absent PLR.^1^, ^12^, ^13^ The sympathetic innervation to the smooth muscle of dilator pupillae is a 3‐neuron pathway. Originating with the first order neuron from the hypothalamus coursing caudally through the tectotegmental tract to the T1‐T3 spinal cord segments where the second order neuron is situated in the gray matter.^1^ From here it courses cranially along the vagus nerve, and at the cranial cervical ganglion the sympathetic trunk separates and enters the cranial cavity with the glossopharyngeal nerve. In the calvarium, the sympathetic trunk enters the orbit along with the ophthalmic nerve branch, finally innervating the orbitalis muscle, third eyelid, smooth ciliary muscle, and the smooth dilator muscle of the pupil in the iris.^1^, ^2^, ^12^ Although lesions along the sympathetic pathway typically cause a miotic pupil and Horner syndrome,^1^, ^2^, ^6^ in rare cases, stimulation of sympathetic fibers can cause Porfour du Petit syndrome resulting in the stimulated site being mydriatic and slightly exophthalmic.^8^ However, in the 2 cases described here, no MRI findings were identified along the pathway of the parasympathetic or sympathetic innervation to the eyes.

Alongside the innervation of the sympathetic and parasympathetic supply to the orbits, cerebellar involvement in pupillary changes has been described.^14^ Previous experimental studies in cats have shown that the cerebellum plays a role in regulating pupil size,^5^, ^15^, ^16^, ^17^, ^18^ but the exact mechanism of this effect is unknown. Experimental ablation of the ipsilateral interposital nucleus in cats has been shown to cause ipsilateral mydriasis, incomplete PLR with ipsilateral widened palpebral fissure, and contralateral third eyelid protrusion. It was however not clear how long the ocular changes lasted.^5^ Additionally, increased ipsilateral extensor tone with decreased or absent postural reactions in the first week, followed by ipsilateral hypermetric gait were noted.^5^ Both dogs had lesions affecting the interposital nucleus, presumed to be the explanation for the observed ipsilateral mydriasis.

Similar clinical signs were described in experimental ablation of the fastigial nucleus including contralateral mydriasis with an incomplete PLR and ipsilateral third eyelid protrusion in cats. Additionally, ipsilateral extensor atonia also was noted after rostral or total experimental ablation of the fastigial nucleus, or contralateral extensor atonia when the caudal pole was removed.^16^, ^19^ Clinical signs improved over 3 weeks in this scenario.^19^ The possibility of the dysfunction of fastigial nucleus involvement was described in a cat with rostral cerebellar artery infarction. However this cat had additional brainstem and forebrain ischemic infarction, and the clinical signs involved bilateral mydriasis with decreased PLRs and absent menace responses bilaterally.^10^ Although these clinical findings are well characterized in experimental cases, the description of these findings in naturally‐occurring cases is lacking.

The pathophysiology of mydriasis with ischemic infarct involving the ipsilateral interposital nucleus has not been definitively explained. In previous experimental cases in cats, evoked potential of the ciliary nerve was recordable after stimulation of the fastigial nucleus, interposital nucleus, and rostral cerebellar peduncle.^15^, ^18^ Additionally, degeneration of the parasympathetic nucleus of the oculomotor nerve and pretectal nucleus was observed after destruction of the fastigial nucleus in experimental cases in cats.^17^ This finding likely indicates a connection between the cerebellum and parasympathetic nuclei of the oculomotor nerve.^15^ However, additional studies are needed to investigate this further.

It is not clear why the first dog had normal ipsilateral palpebral fissure size and the second dog had normal ipsilateral PLR. It is possible that there is no clear demarcation between interposital and fastigial nuclei lesions in naturally‐occurring cases or a different degree of dysfunction was present in these nuclei. More spontaneous cases with lesions in the interposital nucleus are needed to further understand the clinical findings.

Cerebrovascular ischemic infarcts are commonly recognized in dogs,^9^ with the rostral cerebellar artery being most affected.^9^, ^11^, ^20^ Although a definitive histopathologic diagnosis was not made in our cases, the clinical signs and MRI findings were compatible with previously described criteria for cerebellar ischemic infarction.^9^, ^20^ Additionally, both dogs were treated with supportive care and neurological signs improved over a 2‐week period, which would fit with the clinical course of this disease.^9^, ^11^ The second dog had protein‐losing nephropathy as a presumed underlying cause, which was further monitored by the primary care practitioner. The same dog had an additional ischemic infarct located in the caudolateral aspect of the right thalamus and, based on imaging characteristics, 2 chronic infarcts in the right cerebellar hemisphere and the left caudal colliculus.

A variety of cerebellar conditions such as neoplastic,^21^ inflammatory,^22^ infectious,^23^ toxic,^24^ and degenerative^25^ lesions previously have been reported in dogs without naturally‐occurring anisocoria being reported. In a previous report of dogs with suspected rostral cerebellar ischemic infarction, 3 dogs had reported anisocoria but little further information was provided about these cases.^11^

In conclusion, the clinical signs and diagnostic imaging findings in our 2 cases suggest that ischemic infarcts affecting the interposital nucleus can cause reversible ipsilateral mydriasis in dogs.

## CONFLICT OF INTEREST DECLARATION

Authors declare no conflict of interest.

## OFF‐LABEL ANTIMICROBIAL DECLARATION

Authors declare no off‐label use of antimicrobials.

## INSTITUTIONAL ANIMAL CARE AND USE COMMITTEE (IACUC) OR OTHER APPROVAL DECLARATION

Approved by the Royal Veterinary College Social Science Research Ethical Review Board (URN SR2023‐0155).

## HUMAN ETHICS APPROVAL DECLARATION

Authors declare human ethics approval was not needed for this study.
